# Altered KYN/TRP, Gln/Glu, and Met/methionine sulfoxide ratios in the blood plasma of medication-free patients with major depressive disorder

**DOI:** 10.1038/s41598-017-05121-6

**Published:** 2017-07-07

**Authors:** Hidehiro Umehara, Shusuke Numata, Shin-ya Watanabe, Yutaka Hatakeyama, Makoto Kinoshita, Yukiko Tomioka, Kiyoshi Nakahara, Takeshi Nikawa, Tetsuro Ohmori

**Affiliations:** 1 Department of Psychiatry, Graduate School of Biomedical Sciences, Tokushima University, Tokushima, Japan; 2 Center of Medical Information Science, Kochi Medical School, Kochi University, Kohasu, Oko-cho, Nankoku, Japan; 3 Research Institute, Kochi University of Technology, 185 Miyanokuchi, Tosayamada-cho, Kami-shi, Kochi, Japan; 4 Department of Nutritional Physiology, Institute of Medical Nutrition, Tokushima University Graduate School, Tokushima, Japan

## Abstract

Capillary electrophoresis-time-of-flight mass spectrometry (CE-TOFMS) is a comprehensive, quantitative, and high throughput tool used to analyze metabolite profiles. In the present study, we used CE-TOFMS to profile metabolites found in the blood plasma of 33 medication-free patients with major depressive disorder (MDD) and 33 non-psychiatric control subjects. We then investigated changes which occurred in the metabolite levels during an 8-week treatment period. The medication-free MDD patients and control subjects showed significant differences in their mean levels of 33 metabolites, including kynurenine (KYN), glutamate (Glu), glutamine (Gln), methionine sulfoxide, and methionine (Met). In particular, the ratios of KYN to tryptophan (TRP), Gln to Glu, and Met to methionine sulfoxide were all significantly different between the two groups. Among the 33 metabolites with altered levels in MDD patients, the levels of KYN and Gln, as well as the ratio of Gln to Glu, were significantly normalized after treatment. Our findings suggest that imbalances in specific metabolite levels may be involved in the pathogenesis of MDD, and provide insight into the mechanisms by which antidepressant agents work in MDD patients.

## Introduction

Major depressive disorder (MDD) is a common neuropsychiatric disorder with a lifetime prevalence of 16.2%^1^. Although various neurobiological-based hypotheses for the cause of MDD have been proposed^2^, the molecular mechanisms which underlie this disorder remain obscure.

Metabolomics utilizes instruments that can simultaneously quantitate thousands of small molecules in a biological sample^3^. Metabolite levels reflect the final response of an organism to environmental factors, genetic modifications, changes in gut microflora, and altered enzyme kinetic activity. Therefore, individual metabolic state is most closely related to phenotype among other omics technologies, such as genome, transcriptome, and proteome^4^. Capillary electrophoresis-time-of-flight mass spectrometry (CE-TOFMS) is a comprehensive, quantitative, and high throughput tool used to analyze metabolic profiles^5–7^. To date, CE-TOFMS has been used to identify abnormalities of blood metabolites in patients with major depression (MDD), bipolar disorder, schizophrenia, and autism spectrum disorder^8–10^. However, the study which investigated MDD was conducted in a very small cohort of 9 MDD patients and 19 control subjects^9^.

In the present study, we used CE-TOFMS to comprehensively profile metabolites found in the blood plasma of 33 medication-free patients with MDD and 33 non-psychiatric control subjects. Next, we examined correlations which existed between depression severity and the plasma levels of various metabolites. Finally, we followed the patients for 8 weeks to identify any changes which occurred in their metabolite levels.

## Materials and Methods

### Subjects

Thirty-three medication-free patients with MDD were recruited from Tokushima University Hospital in Japan. The diagnosis of MDD was made according to Diagnostic and Statistical Manual of Mental Disorders (DSM-IV) criteria by at least 2 expert psychiatrists on the basis of extensive clinical interviews and a review of medical records. We also followed up 10 patients who received a naturalistic course of treatment with an antidepressant (paroxetine n = 4, duloxetine n = 2, mirtazapine n = 1, or sertraline n = 3). Clinical symptoms were evaluated using the Hamilton Depression Rating Scale (HDRS) at baseline and again after 8 weeks of treatment^11^. Thirty-three age- and sex-matched non-psychiatric healthy control subjects were recruited from hospital staff, students, and company employees who were documented to be free of psychiatric problems and had no past history of mental illness. All participants were of unrelated and of Japanese origin. This study was conducted in accordance with the World Medical Association’s Declaration of Helsinki. The study protocol was approved by the institutional ethics committee of Tokushima University, and all enrolled subjects provided their signed written informed consent for participation.

### Quantitative metabolome analysis

A blood sample was collected from each participant during their morning visit to the hospital. Blood plasma was isolated via centrifugation at 2,000 g for 10 minutes; after which, the plasma-containing supernatant fraction was frozen at −80 °C until further use. 50 μL of plasma sample was added to 450 μL of methanol containing 10 μM each internal standard (internal standard solution: H3304-1002; Human Metabolome Technologies, Inc.). Then, 200 μL of Milli-Q water and 500 μL chloroform were added, and the solution was centrifuged at 2300 g for 5 min at 4 °C. 400 μL of the supernatant was centrifugally filtrated through 5-kDa cut-off filter (UltrafreeMC-PLHCC 250/pk, Human Metabolome Technologies) to remove proteins. The filtrate was centrifugally concentrated in a vacuum evaporator and dissolved in 50 μl of Milli-Q water containing reference compounds before CE-TOFMS analysis. The levels of various metabolites in the plasma samples were measured using a CE-TOFMS system located at Human Metabolome Technologies, Inc. (Yamagata, Japan). Target metabolites were identified by matching their mass-to charge ratio (m/z) values and migration times with the annotation table of the metabolomic library (Human Metabolome Technologies). The relative area for each metabolite was defined as the relative concentration. Details of the procedure of sample preparation and the CE-TOFMS conditions and procedures used to analyze the CE-TOFMS data are described elsewhere^7–9^.

### Statistical analyses

Welch’s t-test was used to compare the levels of each metabolite in the MDD group with its corresponding level in the control group. A false discovery rate (FDR) correction with a significance threshold of 0.05 was applied for multiple testing. Changes which occurred in metabolite levels following treatment with an antidepressant were assessed using a paired t-test. Receiver operating characteristic (ROC) curve analyses of candidate metabolites were performed to discriminate between the MDD and control group, and values for area under the curve (AUC) were obtained. Spearman’s rank correlation coefficient was used to examine relationships between metabolite relative concentrations and clinical symptoms as evaluated by the HDRS. Statistical calculations of Welch’s t-test, paired t-test, and mean ± standard deviation (SD) were carried out using Microsoft Excel 2013 software. ROC curve values and Spearman’s rank correlation coefficients were calculated using R ver 3.2.5. software.

## Results

### Demographics

Some clinical characteristics of the subjects in the two groups are shown in Table 1. There were no significant differences regarding the age and sex of the subjects in the the medication-free MDD group and control group. Depressive symptoms as evaluated by the HDSR were significantly improved after 8 weeks treatment with an antidepressant (mean HDSR score at baseline: 21.0 ± 6.3; mean HDSR score at 8 weeks: 8.5 ± 5.5, p = 0.007; paired t-test).

**Table 1:** Demographic of participants.

		MDD	Control	P
N		33	33	
Male		10	10	
Female		23	23	1
Age		47.1 ± 13.2	46.5 ± 10.1	0.81
HDSR score	Baseline	21.0 ± 6.3		
	8 week	8.5 ± 5.5		

MDD: major depressive disorder; HDRS: Hamilton Depression Rating Scale.

### Differences in metabolites found in the MDD patients and control subjects

**Figure 1:**
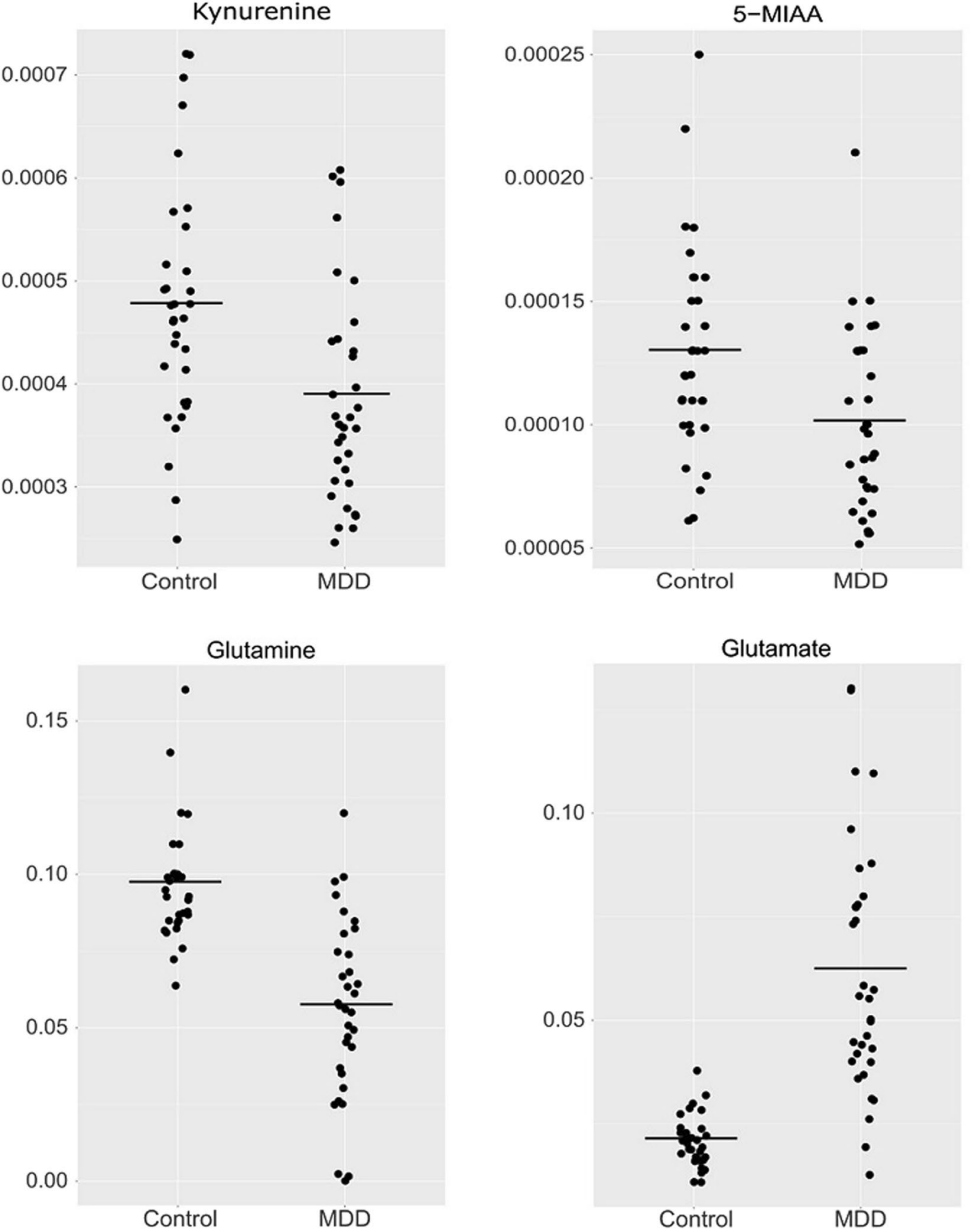
Plasma amino acid levels in medication-free patients with major depressive disorder (MDD) and controls. Y axis represents relative metabolic levels. Bars indicate mean levels in the group. (**a**) Kynurenine were significantly lower in MDD patients than in control subjects (FDR q = 0.0096). (**b**) The levels of 5-Methoxyindoleacetic acid were significantly lower in MDD patients than in control subjects (FDR q = 0.016). (**c**) Glutamate levels were significantly higher in MDD patients than in control subjects (FDR q = 3.7 × 10^−7^). (**d**) Glutamine levels were significantly lower in MDD patients than in control subjects (FDR q = 4.8 × 10^−7^).

A total of 263 candidate peaks (148 cationic compounds and 115 anionic compounds) were detected by CE-TOFMS in our cohort of 66 participants. Among the 263 peaks, 246 peaks were assigned to specific metabolites. 246 metabolite relative concentrations of each individual subject are shown in Supplementary Table S1. Among these 246 metabolites, 106 metabolites, which were present in at least 80% of all subjects (≧53 subjects), were analyzed. Among the 106 metabolites, the levels of 33 metabolites were significantly different in the medication-free MDD group and control group (FDR q < 0.05; Welch’s t-test) (Table 2). Among those 33 metabolites, the levels of 11 metabolites were higher in the MDD group than in the control group. For example, decreased levels of kynurenine (KYN) and 5-Methoxyindoleacetic acid (5-MIAA), which are associated with tryptophan (TRP) metabolism, were found in the MDD group (FDR q = 0.0096 and 0.016, respectively). Increased glutamate (Glu) levels and decreased glutamine (Gln) levels were found in the MDD group (FDR q = 3.7 × 10^−7^ and 4.8 × 10^−7^, respectively) (Fig. 1). This was deemed important, because both Glu and Gln are associated with glutamatergic neurotransmission. Furthermore, the ratios of KYN to TRP, Gln to Glu, and methionine (Met) to methionine sulfoxide in the MDD group were lower than those in the control group (p = 5.4 × 10^−4^, 1.3 × 10^−13^, and 5.6 × 10^−12^, respectively) (Table 3).

**Table 2:** Differences in metabolites in the medication-free MDD patients and control subjects.

Compound name	Control (N = 33)			Medication-free MDD (N = 33)			Medication-free (N = 33) vs Control (N = 33)		
	Mean relative area	S.D.	N	Mean relative area	S.D.	N	p value	FDR q value	AUC value
Cystine	0.0041	0.0016	33	0.0014	0.00096	33	2.7E-11	2.9E-09	0.96
Glutamate (Glu)	0.021	0.0061	33	0.061	0.03	33	6.9E-09	3.7E-07	0.94
Glutamine (Gln)	0.097	0.018	33	0.057	0.029	33	1.5E-08	4.8E-07	0.89
Asparate (Asp)	0.0017	0.00038	33	0.0037	0.0016	33	1.8E-08	4.8E-07	0.91
Inosine monophosphate (IMP)	0.00037	0.0002	32	0.00011	0.000079	25	2.9E-08	6.1E-07	0.93
O-Acetylcarnitine	0.01	0.0021	33	0.0058	0.0036	33	5.6E-08	9.9E-07	0.86
Methionine sulfoxide	0.00049	0.00015	33	0.0028	0.0021	33	5.7E-07	8.6E-06	0.97
3-Phosphoglyceric acid	0.00055	0.00022	33	0.00028	0.00014	23	1.4E-06	1.9E-05	0.84
Isocitric acid	0.0017	0.00037	33	0.0012	0.00041	32	1.1E-05	1.3E-04	0.81
Asparagine (Asn)	0.0086	0.0015	33	0.0063	0.0023	33	1.8E-05	1.9E-04	0.82
Uridine	0.0011	0.00018	33	0.00092	0.00015	33	4.3E-05	4.1E-04	0.77
Phosphorylcholine	0.00029	0.000097	32	0.00044	0.00017	33	5.3E-05	4.7E-04	0.78
Glyceric acid	0.0011	0.00041	33	0.0018	0.00081	33	3.6E-04	0.0029	0.76
Hypoxanthine	0.0031	0.0023	33	0.0012	0.0019	31	4.9E-04	0.0035	0.86
5-Oxoproline	0.0032	0.0006	33	0.0056	0.0035	33	5.3E-04	0.0035	0.74
Methionine (Met)	0.0037	0.00077	33	0.0024	0.0017	27	5.5E-04	0.0035	0.79
Uric acid	0.022	0.0044	33	0.018	0.0056	33	5.6E-04	0.0035	0.73
2-Hydroxybutyric acid	0.003	0.001	33	0.0046	0.0025	33	0.001	0.0056	0.74
cis-Aconitic acid	0.0025	0.00053	32	0.002	0.0006	33	0.001	0.0056	0.71
Creatinine	0.018	0.0032	33	0.016	0.0028	33	0.002	0.0096	0.71
Hippuric acid	0.00064	0.00063	32	0.00025	0.00022	30	0.002	0.0096	0.77
Kynurenine	0.00048	0.00012	33	0.00039	0.0001	33	0.002	0.0096	0.73
S-Methylcysteine	0.00081	0.00031	33	0.00057	0.0003	29	0.003	0.013	0.69
Thiaproline	0.00052	0.00014	32	0.00037	0.00021	32	0.003	0.013	0.73
1-Methyladenosine	0.000059	0.000012	33	0.000049	0.000013	33	0.004	0.016	0.71
5-Methoxyindoleacetic acid (5-MIAA)	0.00013	0.000041	33	0.0001	0.000036	33	0.004	0.016	0.70
Glycerophosphocholine	0.0018	0.00084	33	0.0044	0.0046	33	0.004	0.016	0.63
Glucose 6-phosphate	0.00024	0.00012	33	0.00017	0.000082	28	0.005	0.019	0.71
2-Aminoisobutyric acid	0.0054	0.0014	33	0.0067	0.0022	33	0.009	0.032	0.68
N-Methylnorsalsolinol	0.000085	0.000031	33	0.00011	0.000039	31	0.009	0.032	0.68
Ethanolamine	0.0013	0.00025	33	0.0011	0.00032	33	0.012	0.040	0.70
Threonic acid	0.0023	0.00062	33	0.0029	0.001	33	0.012	0.040	0.65
Isethionic acid	0.0002	0.000063	33	0.00017	0.000028	33	0.014	0.045	0.61

**Table 3:** Ratios of metabolites in control subjects and MDD patients before and after antidepressant treatment.

Ratio	Control (N = 33)		Medication-free MDD (N = 33)		MDD before treatment (N = 10)		MDD after 8 weeks treatment (N = 10)		Medication-free (N = 33) vs Control (N = 33)	Before (N = 10) vs After (N = 10) in MDD
	Mean relative area	S.D.	Mean relative area	S.D.	Mean relative area	S.D.	Mean relative area	S.D.	p value	p value
KYN/TRP	0.031	0.007	0.026	0.005	0.027	0.006	0.031	0.005	5.4E-04	0.074
Glutamine/Glutamate	4.99	1.37	1.5	1.59	1.71	1.57	2.32	1.6	1.3E-13	6.6E-04
Methionine/Methionine sulfoxide	8.13	2.92	2.33	2.19	3.11	2.59	3.86	4.78	5.6E-12	0.61

Next, we performed ROC analyses to examine the power of metabolite relative concentrations to diagnose MDD. An AUC value for each metabolite relative concentration is shown in Table 2. Methionine sulfoxide, an oxidation product of methionine and a possible biomarker of oxidative stress^12^, was able to discriminate medication-free MDD patients from the control subjects, and showed the best AUC among 35 metabolites examined (AUC = 0.97, sensitivity 0.91, and specificity 0.97).

### Relationship between metabolite relative concentrations and depressive symptoms

We examined relationships between the relative concentrations of 106 metabolites and the depressive symptoms of the MDD patients as evaluated by their HDSR scores. We found nominally positive correlations between the relative concentrations of 6 metabolites (isoleucine, leucine, 2-hydroxybutyric acid, valine, 3-hydroxybutyric acid, and 2-aminoadipic acid) and HDSR scores (r = 0.593, r = 0.473, r = 0.421, r = 0.448, r = 0.424, r = 0.367, and r = 0.354, respectively; p < 0.05; Spearman rank correlations). However, only the correlation between a patient’s isoleucine relative concentration and their symptoms reached statistical significance when assessed by multiple comparisons (FDR q < 0.05).

### Metabolite changes following 8 weeks of treatment with antidepressants

Among the 33 metabolites that displayed altered levels in medication-free MDD patients, only two metabolites (Gln and KYN) showed significant changes in their levels after 8 weeks of treatment with antidepressants (Table 4). The levels of both Gln and KYN were significantly increased after treatment (p = 3.3 × 10^−4^ and 3.8 × 10^−3^, respectively; paired-t test). Furthermore, the ratio of Gln to Glu was also significantly increased after treatment (p = 6.6 × 10^−4^). While the KYN to TRP ratio was increased to its control level after treatment, the increase did not reach statistically significance (p = 0.074). On the other hand, the Met to methionine sulfoxide ratio remained unchanged after treatment (p = 0.61).

**Table 4:** The changes in metabolites in the MDD patients after 8 weeks of antidepressant treatment.

Compound name	Medication-free MDD (N = 10)		MDD after 8 weeks treatment (N = 10)		Before (N = 10) vs After (N = 10) in MDD	Medication-on MDD (N = 10) vs CTRL (N = 33)
	Mean relative area	S.D.	Mean relative area	S.D.	p value	p value
Glutamine (Gln)	0.063	0.033	0.085	0.029	3.3E-04	0.25
Kynurenine	0.00042	0.00011	0.00052	0.000095	0.0038	0.23
Uridine	0.00086	0.0001	0.00097	0.00016	0.082	0.047
Hypoxanthine	0.0011	0.0019	0.0026	0.0041	0.087	0.73
3-Phosphoglyceric acid	0.00041	0.00021	0.00036	0.00021	0.092	0.061
Glycerophosphocholine	0.0034	0.0034	0.0013	0.00057	0.1	0.063
Inosine monophosphate (IMP)	0.00012	0.000056	0.00007	0.000021	0.15	7.3E-10
Glutamate (Glu)	0.056	0.025	0.046	0.016	0.16	6.2E-04
Asparate (Asp)	0.0039	0.0011	0.0034	0.0013	0.16	0.0022
Methionine sulfoxide	0.002	0.0019	0.0025	0.0022	0.21	0.021
Creatinine	0.015	0.0033	0.016	0.0027	0.23	0.081
5-Methoxyindoleacetic acid (5-MIAA)	0.00012	0.000041	0.00014	0.000058	0.23	0.5
Glyceric acid	0.0014	0.0005	0.0012	0.00051	0.23	0.89
5-Oxoproline	0.0068	0.0046	0.0052	0.0016	0.24	0.0042
1-Methyladenosine	0.000052	0.000015	0.000058	0.0000085	0.26	0.87
Asparagine (Asn)	0.0072	0.003	0.0079	0.0023	0.31	0.38
Methionine (Met)	0.0027	0.0021	0.0032	0.0024	0.36	0.57
Hippuric acid	0.00024	0.00016	0.00033	0.00019	0.4	0.022
Uric acid	0.019	0.0047	0.02	0.0041	0.4	0.14
Threonic acid	0.0026	0.0007	0.0024	0.00074	0.4	0.69
Ethanolamine	0.0013	0.0004	0.0013	0.0004	0.52	0.98
2-Aminobutyric acid	0.0066	0.0028	0.0061	0.0024	0.54	0.39
N-Methylnorsalsolinol	0.00012	0.000041	0.00011	0.000045	0.61	0.11
2-Hydroxybutyric acid	0.0048	0.003	0.0043	0.0029	0.65	0.17
Isethionic acid	0.00016	0.000034	0.00018	0.000067	0.67	0.39
Phosphorylcholine	0.0004	0.00017	0.00038	0.000095	0.74	0.03
Cystine	0.0015	0.001	0.0016	0.0009	0.76	1.1E-06
S-Methylcysteine	0.00067	0.00022	0.00063	0.0003	0.87	0.13
Thiaproline	0.00041	0.00021	0.0004	0.00017	0.92	0.083
Isocitric acid	0.0015	0.0005	0.0015	0.0004	0.92	0.18
O-Acetylcarnitine	0.0066	0.0029	0.0067	0.0025	0.93	0.0011
cis-Aconitic acid	0.0023	0.0005	0.0023	0.00047	0.95	0.33
Glucose 6-phosphate	0.00017	0.000087	0.00017	0.0001	0.98	0.26

## Discussion

In this study, we used CE-TOFMS to comprehensively profile the metabolites found in blood plasma from medication-free MDD patients and control subjects, and identified novel differential metabolites as well as imbalances of specific metabolite in the plasma of medication-free MDD patients. An organism’s metabolite levels are regarded as its final response to environmental factors, which include treatment with medications. Moreover, several studies have demonstrated that antidepressants influence the levels of certain metabolites^13, 14^. Therefore, we analyzed samples of blood plasma obtained from medication-free MDD patients in an attempt to identify a precise metabolite signature for MDD. Furthermore, we conducted a longitudinal study which examined changes in metabolite abnormalities that occurred after treating MDD patients with antidepressants.

We found that plasma from medication-free MDD patients contained significantly decreased levels of Gln, increased levels of Glu, and had an altered Gln/Glu ratio. However, the abnormal Gln levels and Gln/Glu ratio became significantly normalized after 8 weeks of treatment with antidepressants. In a glutamatergic synapse, the released neurotransmitter is internalized by astrocytes through high affinity sodium-dependent glutamate transporters, and then transformed into Gln by the astrocyte-specific enzyme, glutamine synthetase, and released into the extracellular space, from which it is taken up into neurons through the neutral amino acid transporter and transformed back to Glu by phosphate-activated glutaminase^15^. The complete series of steps from neuronal glutamate release to resynthesis of glutamate from glutamine is called the Glu-Gln cycle^16^, and the Gln/Glu ratio is regarded as a potentially useful index for Glu-Gln cycle in neuronal-glial interactions and the balance of glutamatergic metabolites^17–19^. The abnormal Gln and Glu levels and Gln/Glu ratio found in MDD patients in this study suggest that abnormalities in the Glu-Gln cycle may be involved in the pathogenesis of MDD. Consistent with our results, elevated blood Glu levels in MDD patients^20–24^ as well as decreased plasma Gln in drug-naive MDD patients have been reported^25^. In the current study, the plasma Gln levels in MDD patients were increased after treatment with antidepressants, while the plasma Glu levels following treatment remained unchanged. However, results from other studies have suggested that antidepressant agents might decrease plasma Glu levels in MDD patients^24, 26^. When taken together, the above findings suggest that antidepressant agents may normalize glutamatergic abnormalities by increasing Gln levels and decreasing Glu levels.

We found significantly decreased levels of KYN and 5-MIAA, as well as significantly altered KYN/TRP ratio in the plasma samples from medication-free MDD patients. The abnormal KYN levels were significantly normalized following 8 weeks of treatment with antidepressants. Serotonin is synthesized from ~1% of the available TRP in the body, and TRP is catabolized into KYN by tryptophan 2,3-dioxygenase^27^. 5-MIAA is metabolite formed within the methoxyindole branch of the TRP pathway. The abnormalities of KYN, 5-MIAA, and the KYN/TRP ratio found in this study implicate disruption of the TRP pathway in the pathogenesis of MDD. Consistent with our results, a previous study which used liquid-chromatography-tandem mass spectrometry (LC-MSMS) has reported decreased serum KYN levels in drug-free MDD patients^28^. However, conflicting results have also been reported^29, 30^. These discrepancies between studies may primarily result from whether their respective patient populations received medications, as we found elevated plasma KYN levels and a trend towards elevated KYN/TRP ratio after the patients in our study had been treated with antidepressants. Previous studies have reported elevated serum KYN levels and KYN/TRP ratio during the course of antidepressant treatment in MDD patients^31, 32^.

We found significantly increased levels of methionine sulfoxide, decreased levels of Met, and altered Met/methionine sulfoxide ratio in samples of blood plasma collected from medication-free MDD patients. The abnormal levels of methionine sulfoxide and Met and the abnormal ration of these two metabolites did not significantly change after 8 weeks of treatment with antidepressants. Met is a sulfur-containing essential amino acid, and is used as the first amino acid during the protein translation process. As a result, Met is often a limiting factor for protein synthesis under conditions of Met deficiency^33^. Met also participates in one-carbon metabolism, and decreased levels of Met may be associated with aberrant DNA methylation in MDD^34^. Methionine sulfoxide is a primary oxidation product of Met via its nucleophilic oxidation, and this amino acid can be regarded as a biomarker of oxidative stress^12^. Accumulating evidence implicates oxidative stress in the pathogenesis of MDD^35, 36^. Consistent with our findings, a previous study showed that a 6-week course of antidepressant treatment did not alter the oxidative-antioxidative systems in MDD patients^37^. However, several clinical studies have shown the efficacy of adjunctive treatment with the antioxidant compound, N-acetylcysteine, in MDD patients^38, 39^.

We found a significant positive correlation between isoleucine relative concentration and depression severity. Isoleucine is one of branched-chain amino acids (BCAAs), and this amino acid is transported across the blood-brain barrier as nutrient signals and nitrogen donors in neurotransmitter synthesis and Glu-Gln cycle^40^. Consistent with our findings, depressive-like symptoms in rats following chronic administration of BCAAs^41^ and an association between plasma isoleucine concentration and the severity of MDD have been observed^42^. On the other hand, decreased blood concentrations of BCAAs in patients with MDD treated with antidepressants in comparison with healthy controls and negative correlations between BCAAs concentrations and the severity of MDD have also been reported^43^.

When we compared with our data of MDD with a previous blood study of bipolar disorder using CE-TOFMS^10^, increased Glu levels and decreased cis-Aconitic acid levels, which is an intermediate product of the citric acid cycle, have been commonly observed between studies. These results suggest that not only glutamatergic abnormalities but also abnormalities in the citric acid cycle may be commonly involved in the pathogenesis of mood disorders.

Our study has several limitations that should be mentioned. First, the sample size was relatively small, and larger studies are needed to confirm our results. Second, the patients were not all treated with the same antidepressants. Thus further studies which use specific treatment protocols are required. Third, we examined metabolite levels in samples of peripheral blood gathered from MDD patients. However, those metabolite levels may not have reflected the status of metabolites in the brain tissue of those MDD patients. On the other hand, some of the altered plasma metabolites described in the present study have also been identified in samples of brain tissues and cerebrospinal fluid (CSF). For example, a postmortem study has revealed increased Glu levels in MDD patients^44^. Moreover, proton magnetic resonance spectroscopy (^1^HMRS) studies have demonstrated reduced Gln levels in the hippocampus and pregenual anterior cingulate cortex of unmedicated MDD patients, as well as increased Glu levels in the occipital cortex region^45–47^. Furthermore, abnormality of the Gln/Glu ratio in the CSF of MDD patients and impaired Glu-Gln cycle in the brains of depressed patients have been observed^19, 48^. A high-performance liquid chromatography study has also revealed reduced ethanolamine levels in the CSF of MDD patients^49^. Fourth, we did not take potential confounding factors, such as body mass index and smoking status into consideration in our analysis due to lack of these information. Finally, the blood samples used in our study were collected when the patients visited the hospital in the morning; therefore, the interval between final food intake and blood draw was not unified, and we cannot rule out that food consumption and drinking may have affected the metabolite levels.

In conclusion, we described specific metabolite signatures that were found in samples of blood plasma obtained from medication-free MDD patients. We also conducted a longitudinal study that identified changes which occurred in those signatures after the patients had been treated with antidepressants. Our results suggest that imbalances of specific metabolites may be involved in the pathogenesis of MDD, and provide insight into the mechanisms by which antidepressants affect MDD.

## Electronic supplementary material

Supplementary Table S1
